# Association between different metabolic phenotypes and chronic kidney disease risk: a cohort study

**DOI:** 10.1186/s13098-026-02211-2

**Published:** 2026-06-15

**Authors:** Qi Yu, Haolan Xu, Qin Yang, Wenjing Wang, Ru Zhou, Yanlang Yang

**Affiliations:** 1 Department of Nephrology, Yijishan Hospital, The First Affiliated Hospital of Wannan Medical University, Wuhu, Anhui, 241001 People’s Republic of China; 2 Department of Nephrology, Wanbei Coal-electricity Group General Hospital, Suzhou, 234000 Anhui People’s Republic of China; 3 Nanchang University, Nanchang, 330031 Jiangxi People’s Republic of China

**Keywords:** Chronic kidney disease, Cohort study, Metabolic syndrome, Obesity

## Abstract

**Introduction:**

Metabolic syndrome (MetS) involves conditions that alter renal structure/function; however few studies have considered the dynamic nature of metabolic status when exploring its association with chronic kidney disease (CKD).

**Methods:**

We retrospectively included 7034 participants, categorized into four metabolic phenotypes by BMI and metabolic status: metabolically healthy non-overweight/obesity (MHNO), metabolically healthy overweight/obesity (MHO), metabolically unhealthy non-overweight/obesity (MUNO), and metabolically unhealthy overweight/obesity (MUO). The primary endpoint was incident CKD (eGFR < 60 ml/min/1.73 m² and/or proteinuria for two consecutive years). Associations were assessed using Cox and logistic regression analysis with multivariate adjustments.

**Results:**

The baseline proportions of individuals with MHNO, MUNO, MHO, and MUO phenotypes were 35.6%, 17.5%, 18.3%, and 28.6%, respectively. After multivariate adjustment in Cox analyses, MUNO (HR:1.184; 95%CI:1.031-1.506) and MUO (HR:1.420; 95%CI:1.153-1.749) phenotypes were associated with incident CKD, with no such association in the MHO phenotype. However, in terms of transitions, compared with those with a consistent MHNO phenotype, those with a MHO phenotype evolving to a metabolically unhealthy status had higher incident CKD risk (HR:1.649; 95%CI:1.096-2.482).

**Conclusion:**

Overweight/obesity and metabolic unhealthy status increase CKD risk; individuals with MHO who transition to metabolically unhealthy phenotypes have higher CKD risk than those consistently maintaining MHNO.

**Supplementary Information:**

The online version contains supplementary material available at 10.1186/s13098-026-02211-2.

## Introduction

Metabolic syndrome (MetS), a cluster of interrelated metabolic abnormalities characterized by central obesity, hypertension, dyslipidemia, and insulin resistance [1], has emerged as a global public health crisis affecting approximately one-quarter of the global population [2]. Insulin resistance serves as the core link of MetS, with obesity—especially central obesity—closely associated with its development [3].

Traditional research on MetS has often focused on individual components (e.g., hypertension, insulin resistance) when exploring associations with adverse outcomes, rather than considering integrated metabolic phenotypes [4]. A key distinction lies in metabolic obesity subtypes: metabolically healthy obesity (MHO) is a unique phenotype marked by lower visceral fat mass, reduced ectopic fat accumulation (liver and skeletal muscle), more abdominal subcutaneous adipose tissue, higher insulin sensitivity, and minimal chronic inflammation compared to metabolically unhealthy obesity (MUO) [5]. This difference underscores the need to move beyond static weight-based classifications to dynamic metabolic phenotype assessments.

Obesity and MetS are increasingly recognized as critical factors in chronic kidney disease (CKD) pathogenesis. Obesity, as a complex metabolic disorder, independently predicts new-onset CKD [6], while MetS has been linked to reduced estimated glomerular filtration rate (eGFR < 60 mL/min/1.73 m²) and proteinuria across diverse ethnic populations [7]. CKD carries significant clinical burden, progressing to end-stage kidney disease and increasing cardiovascular event risk [8].

Epidemiological evidence consistently links metabolically unhealthy phenotypes (regardless of weight) to elevated CKD risk, driven by chronic low-grade inflammation, insulin resistance, and dysregulated adipokine secretion—pathways that induce glomerular hyperfiltration, endothelial dysfunction, and renal fibrosis [4, 7]. However, the clinical significance of MHO remains controversial: some studies report no increased CKD risk in stable MHO individuals, while others highlight latent risk associated with long-term metabolic deterioration [4, 7]. This discrepancy emphasizes the critical need to clarify how dynamic transitions between metabolic phenotypes (rather than static classifications) modulate CKD risk, particularly in overweight/obese populations.

Given the global burden of obesity-related metabolic disorders and CKD, resolving these uncertainties is essential for developing targeted prevention strategies. The present study aimed to explore the impact of distinct metabolic phenotypes on CKD risk in a cohort from a physical examination center, and to investigate whether longitudinal changes in these phenotypes influence clinical outcomes.

## Materials and methods

### Study design and participants

A total of 7034 Chinese Han participants with complete health checkup data (2015–2020) were enrolled from the Physical Examination Centre of the First Affiliated Hospital of Wannan Medical University—an affiliated health screening facility of a comprehensive hospital that provides standardized routine health assessments (including anthropometry, laboratory tests, and imaging) for the general population via voluntary appointments or workplace-organized programs.

This study was approved by the Ethics Committee of Yijishan Hospital, Wannan Medical University (Approval Number: 2020-35, Approved in 2020), which specifically authorized the retrospective use of clinical data. All participants provided written informed consent for their health checkup data to be used for research purposes. Annual checkup data were retrieved from the centre’s database for analysis.

Metabolic status was defined in accordance with the 2021 Chinese diagnostic criteria for MetS [1] (derived from the 2020 Edition of the Guidelines for the Prevention and Treatment of Type 2 Diabetes in China, issued by the Chinese Diabetes Society). Due to uncollected waist circumference data, we made a modification to the criteria by excluding waist circumference—justified by previous studies [9] in Chinese populations showing limited additional predictive value of waist circumference when other MetS components are included.

Participants with the following characteristics were excluded: (1) eGFR < 60mL/min/1.73m^2^ at baseline and (2) incompletely documented data on body mass index (BMI), MetS components, and serum creatinine levels. The participants were grouped into the following four metabolic phenotypes: metabolically healthy non-overweight/obesity (MHNO), metabolically healthy overweight/obesity (MHO), metabolically unhealthy non-overweight/obesity (MUNO), and metabolically unhealthy overweight/obesity (MUO). The clinical characteristics of the four groups of subjects at baseline were subsequently described. The primary endpoint was the onset of incident CKD, defined as an eGFR < 60mL/min/1.73m^2^ and/or proteinuria for two consecutive years, in accordance with the criteria and guidelines specified previously [10, 11]. Given that both metabolic and weight status change over time, we additionally observed phenotypic transformation at the end of follow-up. In this study, we initially focused on phenotypic transitions in the MHO group, with the rationale that MUNO and MUO are unfavorable metabolic states, whereas MHNO is considered a favorable metabolic level. We further expanded the analysis to include transitions from MUNO/MUO to metabolically healthy states as suggested. The flow chart is shown in Supplementary Fig. 1.

### Definitions of obesity and metabolic status

A BMI classification system for adults in China was used in the current study, and BMI was calculated as weight (kg)/height squared (m^2^). The measured results were classified as underweight (< 18.5 kg/m^2^), normal weight (18.5–23.9 kg/m^2^), overweight (24–27.9 kg/m^2^), and obesity (≥ 28 kg/m^2^) according to the guidelines and consensus in previous studies [12–14]. All enrolled participants were of Chinese Han ethnicity. Metabolic status was determined using the 2021 Chinese diagnostic criteria for MetS [1], with modifications due to uncollected waist circumference data [9]. Participants with two or more of the following parameters were considered metabolically unhealthy: (1) systolic blood pressure ≥ 130mmHg and/or a diastolic blood pressure ≥ 85mmHg or use of antihypertensive medication; (2) elevated triglyceride concentration > 150 mg/dL; (3) reduced HDL cholesterol concentration < 40 mg/dL in men, and < 50 mg/dL in women; and (4) elevated fasting plasma glucose concentration ≥ 100 mg/dL or use of antidiabetic medications. Finally, based on the above BMI categories and metabolic status (metabolically healthy: not meeting the modified MetS criteria; metabolically unhealthy: meeting the modified MetS criteria), all participants were divided into four metabolic obesity phenotypes with explicit definitions: (1) metabolically healthy non-overweight/obesity (MHNO): Normal weight + metabolically healthy; (2) metabolically healthy overweight/obesity (MHO): Overweight or obesity + metabolically healthy; (3) metabolically unhealthy non-overweight/obesity (MUNO): Normal weight + metabolically unhealthy; and (4) metabolically unhealthy overweight/obesity (MUO): Overweight or obesity +metabolically unhealthy.

### Definition of CKD

The eGFR was calculated using the Chronic Kidney Disease Epidemiology Collaboration (CKD-EPI) equation described by Hoste et al.: 141 × min(Scr/κ, 1)α × max(Scr/κ, 1)-1.209 × 0.993Age × 1.018 [if female], where Scr represents serum creatinine concentration (mg/dL), κ is 0.7 for females and 0.9 for males, α is -0.329 for females and − 0.411 for males, min denotes the minimum of Scr/κ or 1, and max indicates the maximum of Scr/κ or 1 [15]. CKD was defined as an eGFR < 60mL/min/1.73m2 or proteinuria for two consecutive years, as specified in previous guidelines [10, 11].Proteinuria was defined as a semi-quantitative result of ≥ ± for urinary albumin based on the dipstick assay and analyzer criteria.Participants provided early morning urine samples following an overnight fast. Urine routine tests were conducted using commercially available standard reagent strips (dipstick method) coupled with an automated urine analyzer to assess urinary albumin. The test results were independently analyzed and documented by two professional laboratory technicians, who then cross-checked the findings to ensure consistency.

### Statistical analysis

Statistical analyses were performed using SPSS version 26.0 for Windows (SPSS, Chicago, IL, USA). Continuous variables are expressed as mean ± SD. One-way ANOVA was used if continuous variables followed a normal distribution and had homogeneous variance; otherwise, and the Kruskal-Wallis test was used. Categorical variables are presented as numbers (percentages) and were analyzed using the chi-square test. Binary logistic regression and Cox proportional hazard regression models were used to examine the association between metabolic phenotypes and CKD. The risk of incident CKD development was initially analyzed according to baseline metabolic health and obesity, regardless of the transition. In the analysis of the conversion of metabolic phenotypes, participants whose condition was stable in the MHNO group during follow-up were used as the reference group. The time to the development of incident CKD was estimated using the Kaplan-Meier analysis and the log-rank test. Statistical significance was set at a *P*-value of < 0.05.

## Results

### Baseline characteristics of the participants

The baseline characteristics of the participants according to their metabolic phenotypes are presented in Table 1. In total, 7034 individuals were included in the current study. The proportions of individuals with MHNO, MUNO, MHO and MUO phenotypes were 35.6%(*n* = 2507), 17.5% (*n* = 1232), 18.3%(*n* = 1284) and 28.6%(*n* = 2011), respectively. The average age of each group was 44.56, 52.68, 46.83, and 51.72 years. Compared with individuals with MHNO, those with MUO had a more unfavorable metabolic status and worse renal function, which were characterized by higher SBP and higher levels of albumin, fasting glucose, total cholesterol, triglycerides, low-density lipoprotein cholesterol, creatinine, blood urea nitrogen (BUN), and uric acid (UA) but lower estimated eGFR. The metabolic profiles of the participants in the MHO group were better than those in both MUNO and MUO groups. Individuals in the MHO group had lower blood pressure and lower levels of fasting glucose, triglycerides, and low-density lipoprotein cholesterol, but higher eGFRs than those in the MUO and MUNO groups. In the analysis of differences between groups, statistical differences were observed among the four phenotypes (*P* < 0.05) in terms of SBP, DBP, fasting glucose level, triglyceride level, LDL-cholesterol level, eGFR, serum albumin, BUN, creatinine, and UA levels.

**Table 1:** Baseline characteristics of participants

Variables	MHNO	MHO	MUNO	MUO	P value
Participants groups (n,%)	2507(35.6)	1284(18.3)	1232(17.5)	2011(28.6)	-
Demographic characteristics					
Age (years)	44.56 ± 13.03	46.83 ± 12.86	52.68 ± 14.26	51.72 ± 13.35	0.001
Male (n,%)	1133(45.2)	913(71.1)	716(58.1)	1504(74.8)	0.010
Current/former smoker (n, %)	448(17.9)	254(20.6)	377(29.4)	610(30.3)	0.020
Current/former drinker (n, %)	537(22.9)	507(39.5)	368(29.9)	820(40.8)	0.001
Clinical characteristics					
Body mass index (kg/m²)	21.38 ± 1.75	26.12 ± 1.85	22.04 ± 1.46	26.81 ± 2.18	0.001
Systolic BP (mmHg)	108.57 ± 10.66	112.25 ± 9.40^a^	127.04 ± 14.4^a^	123.43 ± 15.55^a^	0.010
Diastolic BP (mmHg)	71.86 ± 6.71	74.47 ± 6.12^a^	82.22 ± 10.66^a^	79.33 ± 9.13^a^	0.001
Laboratory characteristics					
Proteinuria (n, %)	143(5.7)	65(5.1)	87(7.0)	154(7.6)	0.004
Hemoglobin (g/L)	136.44 ± 14.89	134.84 ± 13.82	140.49 ± 14.86^b^	146.27 ± 13.98^b^	0.001
RBC (*10^12^/L)	4.52 ± 0.46	4.73 ± 0.46	4.61 ± 0.46	4.80 ± 0.46	0.020
PLT (*10^9^/L)	185.00 ± 54.96	187.31 ± 54.69	183.05 ± 56.69	188.18 ± 56.95	0.051
Albumin (g/L)	46.86 ± 3.09	46.70 ± 3.05	47.17 ± 3.19	47.23 ± 3.23	0.019
Fasting glucose (mmol/L)	5.25 ± 1.45	5.41 ± 1.12^a^	7.26 ± 2.63^a,b^	7.44 ± 2.79^a^	0.001
Total cholesterol (mmol/L)	4.50 ± 0.91	4.59 ± 0.92	4.73 ± 1.01	4.75 ± 0.96	0.001
Triglyceride (mmol/L)	2.42 ± 1.49	3.40 ± 2.14^a^	3.46 ± 2.08^a,b^	4.39 ± 2.67^a,c^	0.001
HDL-cholesterol (mmol/L)	2.48 ± 0.88	2.31 ± 0.85	2.39 ± 0.91	2.25 ± 0.88	0.001
LDL-cholesterol (mmol/L)	2.31 ± 0.85	2.38 ± 0.85^a,c^	2.42 ± 0.89^a^	2.40 ± 0.88^a,b^	0.001
Alanine transaminase (U/L)	20.25 ± 0.42	29.76 ± 0.64	23.49 ± 0.61	31.73 ± 0.59	0.125
Total bilirubin (U/L)	15.99 ± 6.44	15.97 ± 6.26	15.96 ± 6.30	15.96 ± 6.22	0.989
Blood urea nitrogen (mmol/L)	5.28 ± 1.40	5.59 ± 1.51	5.43 ± 1.46	5.58 ± 1.44	0.001
Creatinine (mmol/L)	64.64 ± 15.29	71.77 ± 21.45	68.18 ± 16.48	72.43 ± 16.44	0.031
Uric acid (mmol/L)	302.12 ± 76.39	355.12 ± 79.81	320.57 ± 81.12	369.21 ± 82.27	0.010
eGFR (mL/min/1.73 m^2^)	86.46 ± 12.16	83.14 ± 12.28^a,c^	81.68 ± 13.23^a,b^	79.90 ± 13.04^a^	0.001

^a^P<0.05, compared with MHNO group, statistical difference was observed. ^b^P<0.05, compared with MHO group, statistical difference was observed. ^c^P<0.05, compared with MUNO group, statistical difference was observed

MHNO= metabolically healthy non-overweight/obesity; MHO= metabolically healthy overweight/obesity; MUNO= metabolically unhealthy non-overweight/obesity; MUO= metabolically unhealthy overweight/obesity; n= number; BP= blood pressure; RBC = red blood cell; PLT= platelet; HDL= high density lipoprotein; LDL = low density lipoprotein; eGFR= estimated glomerular filtration rate

Association between metabolic phenotypes and risks of CKD.

Table 2 presents the results of the subgroup analysis of the association between metabolic phenotype and CKD risk stratified by potential risk factors. The results revealed that the MUO phenotypes consistently exhibited the highest CKD risk across most subgroups. Notably, in the sex-stratified analysis, the increased risk associated with the MUO phenotype was more pronounced in females (adjusted HR: 2.220; 95% CI: 1.207–4.083) than in males (adjusted HR: 1.610; 95% CI: 1.119–2.315), suggesting a potential sex-specific susceptibility to the combined effects of obesity and metabolic unhealthiness. Regarding age, participants in the MUO group aged ≥ 60 years had a significantly elevated CKD risk (HR:1.878; 95%CI: 1.276–2.763), while those aged 36–59 years showed a similar point estimate (HR:1.759; 95%CI: 1.224–2.528), that did not reach statistical significance (*P* > 0.05). In participants with baseline eGFR ranging from 60 to 90 mL/min/1.73m^2^, all three non-MHNO phenotypes were associated with increased CKD risk: MUO (HR:2.116; 95% CI: 1.434–3.123), MUNO (HR: 1.750; 95%CI:1.054–2.906), and MHO (HR: 1.692; 95% CI: 1.165–2.457). Full stratified results are detailed in Table 2.

**Table 2:** Subgroup analysis of the association between metabolic phenotype and CKD risk stratified by potential risk factors

Subgroup	MHNO	MHO	MUNO	MUO	Pvalue
Age(years)					
18–35	1.000(ref)	0.914 (0.355,2.349)	0.798 (0.259,2.461)	0.875 (0.251,3.053)	0.001
36–59	1.000(ref)	1.568 (1.046,2.350)	1.243 (1.023,1.944)	1.759 (1.224,2.528)	0.657
≥ 60	1.000(ref)	1.255 (1.067,2.035)	1.398 (1.091,2.123)	1.878 (1.276,2.763)	0.001
Sex					
Male	1.000(ref)	1.316 (0.930,1.861)	1.586 (0.960,2.620)	1.610 (1.119,2.315)	0.233
Female	1.000(ref)	1.479 (0.839,2.608)	1.238 (0.577,2.653)	2.220 (1.207,4.083)	0.316
Smoking					
Current/former smoker	1.000(ref)	1.368 (1.019,1.837)	1.465 (0.968,2.217)	1.774 (1.302,2.419)	0.538
Non-smoker	1.000(ref)	1.367 (0.979,1.910)	1.251 (0.779,2.010)	1.678 (1.178,2.389)	0.577
Drinking					
Current/former drinker	1.000 (ref)	1.420 (0.826,2.441)	2.383 (1.169,4.858)	1.866 (1.056,3.300)	0.612
Non-drinker	1.000(ref)	1.371 (0.962,1.953)	1.122 (0.669,1.884)	1.754 (1.208,2.547)	0.659
Uric acid					
High	1.000(ref)	1.201 (0.613,2.354)	1.803 (0.759,4.285)	1.627 (0.830,3.191)	0.001
Normal/Low	1.000(ref)	1.408 (1.013,1.957)	1.308 (0.804,2.129)	1.729 (1.212,2.465)	0.545
Baseline eGFR(mL/min per 1.73 m2)					
60– 89	1.000(ref)	1.692 (1.165,2.457)	1.750 (1.054,2.906)	2.116 (1.434,3.123)	0.001
≥ 90	1.000(ref)	0.921 (0.558,1.519)	0.983 (0.467,2.066)	1.234 (0.719,2.117)	0.420

P-value for interaction between subgroup and metabolic phenotypes.Adjusted for covariates included age, sex, lifestyle factors (smoking and alcohol drinking), uric acid, serum creatinine and baseline eGFR

MHNO=metabolically healthy non-overweight/obesity; MHO=metabolically healthy overweight/obesity; MUNO=metabolically unhealthy non-overweight/obesity; MUO=metabolically unhealthy overweight/obesity; n=number; BP=blood pressure; eGFR=estimated glomerular filtration rate

### Longitudinal association and survival analysis

The five-year follow-up data indicated that an increase in BMI was positively associated with the risk of CKD (Table 3). Patients with MetS have increased risk of CKD compared to those with normal metabolism. Among the four metabolic phenotypes, the risk of developing CKD was highest in the MUO group (*P* < 0.001).

**Table 3:** Multivariable-adjusted odds ratios of CKD by BMI-MetS categories

	Odds Ratios (95% CI)					
	Model 1	P-value	Model 2	P-value	Model 3	P-value
BMI status						
Under/Normal weight(< 24kg/m^2^)	1.000 (ref)	-	1.000 (ref)	-	1.000 (ref)	-
Overweight(24 -27.9kg/m^2^)	1.377 (1.151,1.648)	< 0.001	1.381 (1.154,1.653)	< 0.001	1.300 (1.075,1.572)	< 0.001
Obesity(≥ 28kg/m^2^)	1.543 (1.181,2.016)	0.001	1.563 (1.195,2.044)	0.001	1.413 (1.063,1.879)	< 0.001
MetS status						
Non-MetS	1.000 (ref)	-	1.000 (ref)	-	1.000 (ref)	-
MetS	1.652 (1.390,1.962)	< 0.001	1.651 (1.389,1.963)	< 0.001	1.593 (1.326,1.913)	< 0.001
BMI-MetS phenotype						
MHNO	1.000 (ref)	-	1.000 (ref)	-	1.000 (ref)	-
MHO	1.250 (1.095,1.630)	0.010	1.258 (1.064,1.641)	0.009	1.172 (1.089,1.547)	0.026
MUNO	1.268 (1.098,1.636)	0.007	1.266 (1.080,1.636)	0.007	1.218 (1.031,1.594)	0.015
MUO	1.755 (1.408,2.187)	< 0.001	1.762 (1.413,2.196)	< 0.001	1.615 (1.279,2.038)	< 0.001

Model 1: adjusted for age, sex

Model 2: adjusted for covariates in Model 1 plus lifestyle factors (smoking and drinking)

Model 3: adjusted for covariates in Model 2 plus uric acid, creatinine and baseline estimated glomerular filtration rate

CKD= chronic kidney disease; BMI= body mass index; MetS= metabolic syndrome; MHNO=metabolically healthy non-overweight/obesity; MHO=metabolically healthy overweight/obesity; MUNO=metabolically unhealthy non-overweight/obesity; MUO=metabolically unhealthy overweight/obesity; ref= reference

**Fig. 1:**
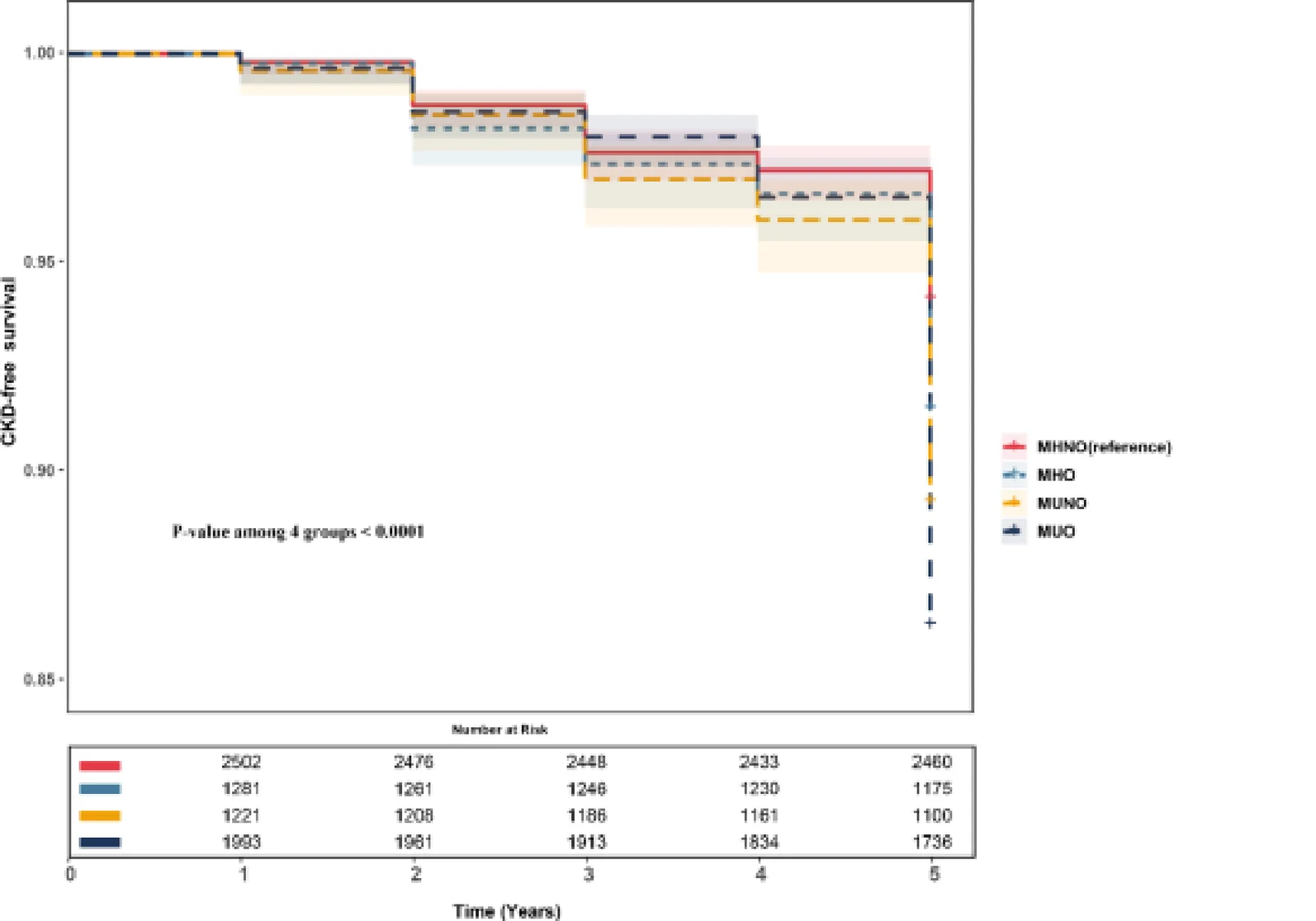
Cumulative curves of CKD-free survival in four baseline phenotypes. CKD= chronic kidney disease; MHNO= metabolically healthynon-overweight/obesity; MHO= metabolically healthy overweight/obesity; MUNO= metabolically unhealthy non overweight/obesity; MUO= metabolically unhealthy overweight/obesity

The cumulative curves of CKD-free survival for the four baseline phenotypes (Fig. 1**)** revealed that the risk of developing CKD was greater in the other three groups than in the MHNO group (*P* < 0.001). Table 4 presents the Cox regression results for the association between metabolic phenotypes and CKD. After multivariate adjustment, the MUNO (HR:1.184; 95%CI: 1.031–1.506) and MUO groups (HR: 1.420; 95% CI: 1.153–1.749) were still associated with the occurrence of CKD, but no such correlation was detected in the MHO group.

**Table 4:** Univariate Cox regression analysis on the association between each phenotype and CKD

	MHNO	MHO	MUNO	MUO
N(% of total)	2507(35.6)	1284 (18.3)	1232 (17.5)	2011 (28.6)
Number of CKD event（%）	147(22.2%)	109 (16.4%)	132 (19.9%)	275 (41.5%)
Crude HR (95% CI)	1(ref)	1.455 (1.135,1.863)	1.844 (1.458,2.332)	2.342 (1.917,2.862)
P-value	-	0.003	< 0.001	< 0.001
Multivariate-adjusted HR (95% CI)	1(ref)	1.059 (0.819,1.369)	1.184 (1.031,1.506)	1.420 (1.153,1.749)
P-value	-	0.664	0.017	< 0.001

Adjusted for covariates included age, sex, lifestyle factors (smoking and drinking), uric acid, creatinine and baseline estimated glomerular filtration rate

CKD=chronic kidney disease; MHNO=metabolically healthy non-overweight/obesity; MHO=metabolically healthy overweight/obesity; MUNO=metabolically unhealthy non-overweight/obesity; MUO=metabolically unhealthy overweight/obesity; N= number; HR= hazard ratio; ref= reference

Risk of incident CKD by MHO transition.

Given that metabolic status and BMI would change over time, we further examined phenotypic transitions among participants with the MHO phenotype during follow-up. Among MHO individuals, 59.6% underwent a phenotypic transition:28.6% converted to MHNO, 1.7% to MUNO, and 29.3% to MUO. The remaining 40.4% maintained a stable MHO phenotype throughout the study period. Compared with participants who remained in the MHNO group, participants with MHO who converted to the MUO phenotype had an increased risk of developing CKD (HR:1.649; 95% CI: 1.096–2.482; Table 5).Supplementary Table 1 supplements presents the univariate Cox regression analysis of the association between individual variables and incident CKD.

**Table 5:** Incident risk for CKD according to the transition for the obese metabolic phenotypes

Baseline	Last follow-up	Participants, (n,%)	Odds Ratios (95% CI)	
			Model 3	P value
MHNO	MHNO	1825(72.8%)	1.000(Ref)	-
	MHO	291 (11.6%)	1.057(0.627,1.784)	0.835
	MUNO	221(8.9%)	1.328(0.754,2.312)	0.468
	MUO	170(6.8%)	0.663(0.229,1.902)	0.441
MHO	MHNO	119(9.3%)	1.166(0.551,2.470)	0.688
	MHO	755(58.8%)	0.967(0.688,1.401)	0.861
	MUNO	28(2.2%)	0.569(0.074,4.367)	0.588
	MUO	382(29.8%)	1.649(1.096,2.482)	0.016
MUNO	MHNO	642(52.2%)	0.805(0.607,1.068)	0.733
	MHO	94(7.6%)	0.584(0.535,0.873)	0.133
	MUNO	356(28.9%)	0.682(0.386,0.679)	0.287
	MUO	138(11.2%)	0.551(0.457,0.664)	0.268
MUNO	MHNO	185(9.2%)	0.574(0.432,0.763)	0.371
	MHO	743(36.9%)	0.713(0.558,0.912)	0.558
	MUNO	91(4.5%)	0.830(0.625,1.102)	0.625
	MUO	992(49.3%)	1.415(0.946,1.748)	0.564

Model 3: adjusted for covariates in age, sex, lifestyle factors (smoking and drinking), uric acid, creatinine and baseline estimated glomerular filtration rate

MHNO=metabolically healthy non-overweight/obesity; MHO=metabolically healthy overweight/obesity; MUNO=metabolically unhealthy non-overweight/obesity; MUO=metabolically unhealthy overweight/obesity

## Discussion

Existing evidence confirms the association between metabolic syndrome (MetS) and chronic kidney disease (CKD) risk, but the mechanisms linking metabolic phenotypes to CKD remain unclear. Our large retrospective cohort study found that overweight/obese individuals with metabolic abnormalities face elevated CKD risk, and dynamic analysis of phenotypic evolution revealed that metabolically healthy obesity (MHO) is involved in CKD development—specifically through transition to unhealthy states rather than the stable MHO phenotype itself.

MetS encompasses abdominal obesity, type 2 diabetes, hypertension, and dyslipidemia, with MHO representing a unique subtype characterized by favorable metabolic profiles and better outcomes than metabolically unhealthy phenotypes. Consistent with previous studies reporting no increased CKD risk in stable MHO individuals [16, 17], our findings confirm that stable MHO does not confer elevated CKD risk in the absence of phenotypic transition. However, other research has linked metabolically healthy overweight/obesity to higher CKD risk than normal weight [18, 19], and our data clarify this discrepancy by identifying phenotypic progression to metabolic unhealthiness as the key driver of increased risk.

Obesity plays an important role in the MHO-CKD association [20, 21], with shared pathophysiological pathways underlying this link: (1) Glomerular hyperfiltration: obesity increases renal blood flow and glomerular pressure, leading to glomerular hypertrophy and progressive injury [22]; (2) Activation of the renin–angiotensin–aldosterone system (RAAS): adipose tissue secretes renin, activating RAAS to promote sodium retention, hypertension, and renal fibrosis [23]; (3) Ectopic fat deposition: peri-renal fat accumulation causes mechanical compression, reduced renal perfusion, and local inflammation [24]; (4) Obstructive sleep apnea: common in obesity, leading to intermittent hypoxia, increased oxidative stress, and endothelial dysfunction [25]; (5) Hyperuricemia: obesity-induced hyperuricemia stimulates RAAS, promotes inflammation, and impairs endothelial function [26]; (6) Insulin resistance: increases renal sodium reabsorption, contributes to hypertension, impairs renal vasodilation, and induces glomerular/podocyte injury [22]; (7) Adipokine dysregulation: excess leptin and leptin resistance promote sympathetic activation, oxidative stress, and direct glomerular hypertrophy; (8) Systemic inflammation: obesity-induced chronic low-grade inflammation (e.g., increased TNF-α, IL-6) damages renal tissue. These pathways converge to endothelial dysfunction and renal fibrosis, ultimately leading to CKD.

Our analysis indicated that, compared with males, females had a higher risk of developing CKD in the MUO group. Several factors are likely responsible for the sex differences, for instance, sex hormone-binding globulin (SHBG), which regulates sex hormone levels and activity, is associated with a lower risk of CKD and better kidney function in men than in women [27]. In addition, previous studies have shown that females have higher free fatty acid storage rates than males [21, 27–33]. Free fatty acids are harmful to the kidneys, and higher free fatty acid levels are correlated with the progression of CKD [34, 35].

Many studies have investigated the links among obesity, MetS, and CKD; however few longitudinal studies have investigated metabolic phenotype transitions [36, 37]. Our study included complete clinical data and revealed the dynamic transformation between metabolic phenotypes. We also revealed that, even among patients with MHNO phenotypes without risk factors at the beginning of follow-up, nearly 25% of MHNO subjects transitioned to other unhealthy metabolic phenotypes after 5 years of follow-up. These findings demonstrate that the dynamic transformation between metabolic phenotypes eventually leads to an increased risk of CKD over time.

However, while we used the metabolically healthy non-obese (MHNO) phenotype as the universal reference to assess absolute risk, we acknowledge that using a stable MHO group as the reference would provide a more direct estimate of the risk associated specifically with transitioning from MHO to an unhealthy state. Although our current data indirectly support that metabolic deterioration drives the increased risk, future studies with larger sample sizes of MHO individuals are warranted to perform such subgroup analyses and futher confirm our findings.

A minor proportion of participants had proteinuria at baseline (5.1%–7.6%); however, our strict outcome definition required consecutive-year abnormalities, which minimized potential outcome misclassification, and these participants were retained to preserve cohort completeness and statistical power.

In summary, we found an increased risk of CKD in individuals with MHO, MUNO, and MUO phenotypes compared to those with the MHNO phenotype. Notably, the metabolic state is a dynamic condition, and the MHO phenotype can transform into an unhealthy metabolic phenotype, leading to a high risk of developing CKD.

## Conclusion

Both overweight/obesity and metabolically unhealthy status can lead to increased risk of developing CKD, and compared with the group that consistently maintained the MHNO phenotype during follow-up, individuals in the MHO group are at increased risk of CKD.

## Supplementary Information

Below is the link to the electronic supplementary material.

Supplementary Material 1.

Supplementary Material 2.

## Data Availability

All data generated or analyzed during this study are included in this article and its online supplementary material files. Further enquiries can be directed to the corresponding author. The data underlying this article will be shared on reasonable request to the corresponding author.
