# Supplementary material for: Association between different metabolic phenotypes and chronic kidney disease risk: a cohort study
Source: Diabetol Metab Syndr. 2026 Jun 15;18:181. doi: 10.1186/s13098-026-02211-2 (PMC13501839; doi:10.1186/s13098-026-02211-2)
Supplement: Supplementary file 1 [file 13098_2026_2211_MOESM1_ESM.docx]

Supplementary Figure 1. Flow diagram of the screening and enrollment of study participants.

Abbreviations: eGFR=estimated glomerular filtration rate. MHNO=metabolically healthy non-overweight/obesity; MHO=metabolically healthy overweight/obesity; MUNO=metabolically unhealthy non-overweight/obesity; MUO=metabolically unhealthy overweight/obesity.
