# Supplementary material for: Association between different metabolic phenotypes and chronic kidney disease risk: a cohort study
Source: Diabetol Metab Syndr. 2026 Jun 15;18:181. doi: 10.1186/s13098-026-02211-2 (PMC13501839; doi:10.1186/s13098-026-02211-2)
Supplement: Supplementary file 2 [file 13098_2026_2211_MOESM2_ESM.docx]

| **Supplementary table 1. Univariate Cox regression analysis on the association between variables and CKD.** | | |
| --- | --- | --- |
| **Variables** | ***P* value** | **OR (95%CI)** |
| **Age(year)** | **0.001** | 1.122(1.030-1.233) |
| **Sex** |  |  |
| Male |  | 1.000(ref) |
| Female | **0.012** | 1.078(1.021-1.843) |
| **Smoking** |  |  |
| Non-smoker |  | 1.000(ref) |
| Current/former smoker | 0.042 | 0.381(0.116-0.925) |
| **Drinking** |  |  |
| Non-drinker |  | 1.000(ref) |
| Current/former drinker | 0.057 | 0.427(0.225-1.233) |
| **Uric acid** |  |  |
| Normal/Low |  | 1.000(ref) |
| High | 0.061 | 0.233(0.182-1.208) |
| **Baseline eGFR**  **< 90 mL/min per 1.73 m^2^** | **0.001** | 1.158(1.048-2.352) |
| Abbreviations: MHNO=metabolically healthy non-overweight/obesity; MHO=metabolically healthy overweight/obesity; MUNO=metabolically unhealthy non-overweight/obesity; MUO=metabolically unhealthy overweight/obesity; eGFR=estimated glomerular filtration rate;OR=odd ratio; CI=confidence interval. | | |
